# Identification of a five‐mRNA signature as a novel potential prognostic biomarker in pediatric Wilms tumor

**DOI:** 10.1002/mgg3.1032

**Published:** 2019-11-07

**Authors:** Xiao‐Dan Lin, Yu‐Peng Wu, Shao‐Hao Chen, Xiong‐Lin Sun, Zhi‐Bin Ke, Dong‐Ning Chen, Xiao‐Dong Li, Yun‐Zhi Lin, Yong Wei, Qing‐Shui Zheng, Ning Xu, Xue‐Yi Xue

**Affiliations:** ^1^ Departments of Urology the First Affiliated Hospital of Fujian Medical University Fuzhou China

**Keywords:** bioinformatics, biomarkers, mRNAs, prognosis, Wilms tumor

## Abstract

**Background:**

The aim of this study was to generate a prognostic model to predict survival outcome in pediatric Wilms tumor (WT).

**Methods:**

The data including mRNA expression and clinical information of pediatric WT patients were downloaded from the Therapeutically Available Research to Generate Effective Treatments (TARGET) database. The differentially expressed genes were identified and a prognostic signature of pediatric WT was generated according to the results of univariate and multivariate Cox analysis. Receiver operating characteristic (ROC) curve was used to evaluate the five‐mRNA signature in pediatric Wilms tumor patients. Bootstrap test with 500 times was used to perform the internal validation.

**Results:**

We identified 6,964 differentially expressed mRNAs associated with pediatric WT, including 3,190 downregulated mRNAs and 3,774 up‐regulated mRNAs. Univariate and multivariate Cox analysis identified five mRNAs (*SPRY1*, *SPIN4*, *MAP7D3, C10orf71*, *and SPAG11A*) to establish a predictive model. The risk score formula is as follows: Risk score = 0.3036*SPIN4 + 0.8576*MAP7D3 −0.1548*C10orf71 −0.7335*SPRY1 −0.2654*SPAG11A. The pediatric WT patients were divided into low‐risk group and high‐risk group based on the median risk score (value = 1.1503). The receiver operating characteristic (ROC) curve analysis revealed good performance of the 5‐mRNA prognostic model (the area under the curve [AUC] was 0.821). Bootstrap test (Bootstrap resampling times = 500) was used to perform the internal validation and revealed that the AUC was 0.822. REACTOME, KEGG, and BIOCARTA pathway analyses demonstrated that these survival‐related genes were mainly enriched in ErbB2 and ErbB3 signaling pathways, and calcium signaling pathway.

**Conclusion:**

The five‐mRNA signature can predict the prognosis of patients with pediatric WT. It has significant implication in the understanding of therapeutic targets for pediatric WT patients. However, further study is needed to validate this five‐mRNA signature and uncover more novel diagnostic or prognostic mRNAs candidates in pediatric WT patients.

## Graphical abstract

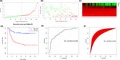

## 1 INTRODUCTION

The incidence of Wilms tumor (WT) among children younger than 15 years is 7.1 cases per 1 million. WT, which accounts for more than 90% of renal tumors among pediatric patients, is the most common solid renal malignancy (Cone et al., 2016; Pastore et al., 2006; Stokes et al., 2018). The incidence is lower in Asians when compared with that in the United States. The male to female ratio is quite different between unilateral cases and bilateral cases of WT (0.92–1.00 in unilateral cases and 0.6–1.00 in bilateral cases). Also, the mean age at diagnosis between unilateral cases and bilateral cases are different (44 months in unilateral cases and 31 months in bilateral cases) (Breslow, Olshan, Beckwith, & Green, 1993; Phelps et al., 2019; Wang, Lou, & Ma, 2019). Scott RH et al. (Scott, Stiller, Walker, & Rahman, 2006) reported that about 10% of children with WT was suffering from congenital malformation syndrome. WT arise after a limited number of genetic aberrations, as reported by the Gadd S et al. (Gadd et al., 2017), including *Wilms tumor 1 transcription factor* (*WT1*; OMIM: 194070), *catenin beta 1* (*CTNNB1*; OMIM: 116806), or *APC membrane recruitment protein 1* (*AMER1*; OMIM: 300647) etc. Recently, some studies(Moch, Cubilla, Humphrey, Reuter, & Ulbright, 2016; Wegert et al., 2015) have revealed that approximately 15% of WT have microRNA‐processing gene mutations, including *drosha ribonuclease III* (*DROSHA*; OMIM: 608828), *DGCR8 microprocessor complex subunit* (*DGCR8*; OMIM: 609030), *dicer 1 ribonuclease III* (*DICER1*; OMIM: 606241), *exportin 5* (*XPO5*; OMIM: 607845) and *TARBP2 subunit of RISC loading complex* (*TARBP2*; OMIM: 605053).

Besides stage and histology, a variety of clinical and biological factors was used to define treatment, including age, tumor size and volume, the loss of heterozygosity at chromosomes 1p and 16q, and response to chemotherapy (Dome et al., 2013; Dome, Perlman, & Graf, 2014). The treatment studies of children with WT have been evaluated by two different clinical groups, including COG Renal Tumor Committee (COG RTC) (D'Angio et al., 1989) and SIOP (Graf, Tournade, & de Kraker, 2000). The standard approach to WT treatment in the COG RTC group was immediate surgery, while the first step in treatment in SIOP was preoperative chemotherapy. Postoperative chemotherapy was used in both groups. The long‐term survival outcomes of pediatric WT patients has improved gradually in the recent years, however, the subsequent chronic health conditions, including renal failure, cardiac toxicity, and subsequent malignancies should not be ignored (Aldrink et al., 2018; Gratias et al., 2016; Wong et al., 2016).

High risk groups compose 25% of patients with WT, including those with unfavorable histological, bilateral disease, and recurrence disease (Dome et al., 2015). As we all know, the heterogeneity among individuals often makes conventional prognostic systems. For instance, the risk stratification of TNM staging system is not sufficient. Besides, it is also insufficient to provide an accurate estimation of survival outcome. Thus, it is urgent to generate an accurate prognostic model to predict the survival outcomes in pediatric WT patients. Prognostic model plays a crucial role in the management of tumors, such as prostate specific antigen, alpha fetoproteinca, and carcinoembryonic antigen. Although a meta‐analysis (Cone et al., 2016) reported that a large number of tumor biomarkers have been used to predict the prognostic outcomes in pediatric WT, there has been no prior study which has focused on an mRNA signature to predict the prognosis of WT patients.

The present study aimed to conduct an integrated study to develop a five‐mRNA signature for the prognostic predication of WT patients by analyzing pediatric WT patients from Therapeutically Available Research to Generate Effective Treatments (TARGET) database.

## 2 MATERIALS AND METHODS

### 2.1 Acquisition of TARGET pediatric WT data

The RNA‐seq data (level 3) and corresponding clinical information of pediatric WT in TARGET database were downloaded from Genomic Data Commons Data Portal (portal.gdc.cancer.gov/). We identified 136 cases investigated in this study, including 6 normal samples and 130 WT samples. No further normalization was needed for the expression data downloaded from TARGET database which have already been normalized. The data with no expression were deleted previously. The level 3 RNA‐seq data between normal tissues and WT tissues were analyzed by edgeR package based on R language for differential expression analysis. Genes with absolute log 2 fold change > 1 and *p* < .05 were regarded as differentially expressed mRNAs. Since the data come from the TARGET database, no further approval was required from the Ethics Committee.

### 2.2 Survival analysis

Clinical data were combined with those pediatric patients with WT in TARGET database to identify the prognostic differential expressed mRNAs signature. The survival curves of those samples with differential expressed mRNAs were plotted by using the “survival” package in R. The primary endpoint was overall survival. Univariate Cox analysis and multivariate Cox analysis were performed in this study. All identified differential expressed mRNAs were performed by univariate Cox analysis. The hazard ratio and P value of all differential expressed mRNAs were calculated. Receiver operating characteristic (ROC) curve has been used to prove the sensitivity and specificity of the calculated riskscore in predicting the overall survival of pediatric WT patients. The area under the curve (AUC) was generated and bootstrap was used to estimate 95%CI with the AUC.

### 2.3 Pathway analysis

The DAVID online tool (https://david.ncifcrf.gov/) was used to annotate the survival‐related mRNAs as previously described (Ke et al., 2019; Xu et al., 2019; Xu, Wu, Yin, Xue, & Gou, 2018). REACTOME (http://www.reactome.org/), Kyoto Encyclopedia of Genes and Genomes (KEGG) (http://www.genome.jp/kegg/pathway.html), and BIOCARTA (https://cgap.nci.nih.gov/Pathways/BioCarta_Pathways) pathway databases were used to perform pathway analyses among survival‐related mRNAs screened by univariate Cox analysis.

### 2.4 Statistical analysis

Kaplan–Meier survival analyses were used to determine the overall survival of pediatric Wilms patients who were classified as high expression and low expression group based on the median expression level of each differentially expressed mRNA. Log‐rank test with the R package “survival” was used to determine the difference in the survival of pediatric patients. *p* < .05 was considered as statistically significant.

## 3 RESULTS

### 3.1 Survival analysis by Kaplan–Meier method among differentially expressed mRNAs in pediatric WT patients

**Figure 1:**
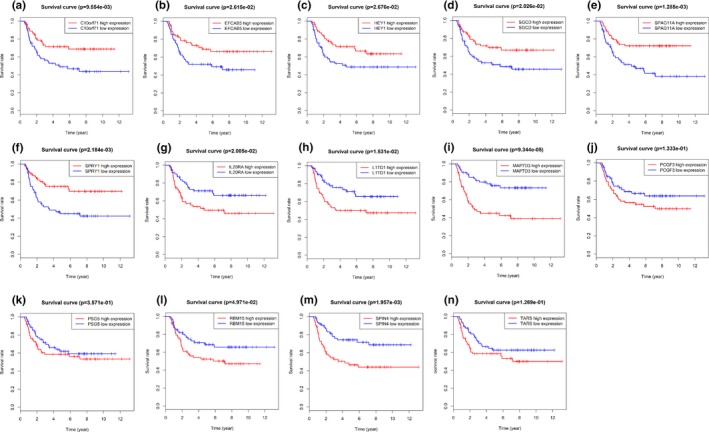
Kaplan–Meier survival curve analysis for overall survival of mRNAs in pediatric Wilms tumor patients. (a–f) mRNAs were associated with favor overall survival in pediatric patients with WT (*p* < .05). (g–n) mRNAs were associated with worse overall survival in pediatric patients with WT (*p* < .05)

We identified 6,964 differentially expressed mRNAs, including 3,190 downregulated mRNAs and 3,774 upregulated mRNAs. Survival analyses among each deferentially expressed mRNAs were performed by Kaplan–Meier method subsequently. The high expression and low expressed of those genes including *chromosome 10 open reading frame 71 *(*C10orf71*), *EF‐hand calcium binding domain 5 *(*EFCAB5*), *hes‐related family bHLH transcription factor with YRPW motif 1*(*HEY1*; OMIM: 602953), *interleukin 20 receptor subunit alpha* (*IL20RA*; OMIM: 605620), *LINE1 type transposase domain containing 1*(*L1TD1*), *MAP7 domain containing 3* (*MAP7D3*; OMIM: *300930*), *polycomb group ring finger 3* (*PCGF3*; OMIM: 617543), *pregnancy specific beta‐1‐glycoprotein 5* (*PSG5*; OMIM: 176394), *RNA binding motif protein 15* (*RBM15*; OMIM: 606077), *sarcoglycan delta* (*SGCD*; OMIM: 601411), *sperm associated antigen 11A* (*SPAG11A*), *spindlin family member 4* (*SPIN4*), *sprouty RTK signaling antagonist 1* (*SPRY1*; OMIM: 602465), *threonyl‐tRNA synthetase* (*TARS*). The high expression of mRNAs, including *C10orf71*, *EFCAB5*, *HEY1*, *SGCD*, *SPAG11A* and *SPRY1* were associated with favor overall survival in pediatric patients with WT (*p* < .05) (Figure 1a–f). Plus, the low expression of mRNAs, including *IL20RA*, *L1TD1*, *MAP7D3*, *PCGF3*, *PSG5*, *RBM15*, *SPIN4*, and *TARS* were associated with worse overall survival in pediatric patients with WT (*p* < .05) (Figure 1g–n).

### 3.2 Survival analysis by univariate cox analysis and multivariate cox analysis among differentially expressed mRNAs in pediatric WT patients

Univariate Cox analysis for all differentially expressed mRNAs was assessed to determine the survival‐related mRNAs (Table S1). The primary endpoint for survival analysis was overall survival. The significant level cutoff threshold was set as 0.001 (*p* < .001) to identify the candidate mRNAs (Table 1). Multivariate Cox analysis was then performed by using these candidate mRNAs identified by univariate Cox analysis. Finally, five mRNAs (*SPRY1*, *SPIN4*, *MAP7D3, C10orf71*, and *SPAG11A*) were identified (Table 1). The results of multivariate Cox analysis also revealed the independent prognostic value of these 5 hub mRNAs. Two were associated with high risk of death in pediatric WT (*SPIN4* and *MAP7D3*). *SPIN4* and *MAP7D3* were associated with a poor overall survival of pediatric WT patients. Specifically, the risk of death in patient with high expression of *SPIN4* was 1.355 times higher than patient with low expression of *SPIN4*. Plus, the risk of death in patient with higher expression of *MAP7D3* was 2.358 times higher than patient with low expression of *MAP7D3*. Also, three were associated with low risk of death in pediatric WT (*SPRY1*, *C10orf71*, and *SPAG11A*). the risk of death in patients with low expression of *SPRY1* was 2.083 times higher than patient with high expression of *SPRY1*. Plus, the risk of death in patients with low expression of *C10orf71* was 1.167 times higher than patients with high expression of *C10orf71*. Also, the risk of death in patients with low expression of *SPAG11A* was 1.304 times higher than patients with high expression of *SPAG11A*.

**Table 1:** Univariate and multivariate Cox analysis of overall survival

Univariate Cox analysis			Multivariate Cox analysis		
mRNA	HR	*p*	mRNA	coef	HR
*SPRY1*	0.354022	6.96E‐07	*SPRY1*	−0.733483894	0.480232991
*SPIN4*	2.035141	2.51E‐05	*SPIN4*	0.303637441	1.35477778
*IL20RA*	1.295478	0.000108			
*EFCAB5*	0.442097	0.000172			
*PSG5*	1.525531	0.000218			
*TARS*	4.606025	0.000239			
*MAP7D3*	4.359476	0.000297	*MAP7D3*	0.857624458	2.357553568
*C10orf71*	0.797138	0.000337	*C10orf71*	−0.154814358	0.856574179
*SPAG11A*	0.694749	0.000457	*SPAG11A*	−0.265447689	0.766862557
*HEY1*	0.608125	0.000547			
*SGCD*	0.716995	0.000745			
*L1TD1*	1.258341	0.000811			
*PCGF3*	2.460722	0.000848			

### 3.3 The development of the 5‐mRNA prognostic model

**Figure 2:**
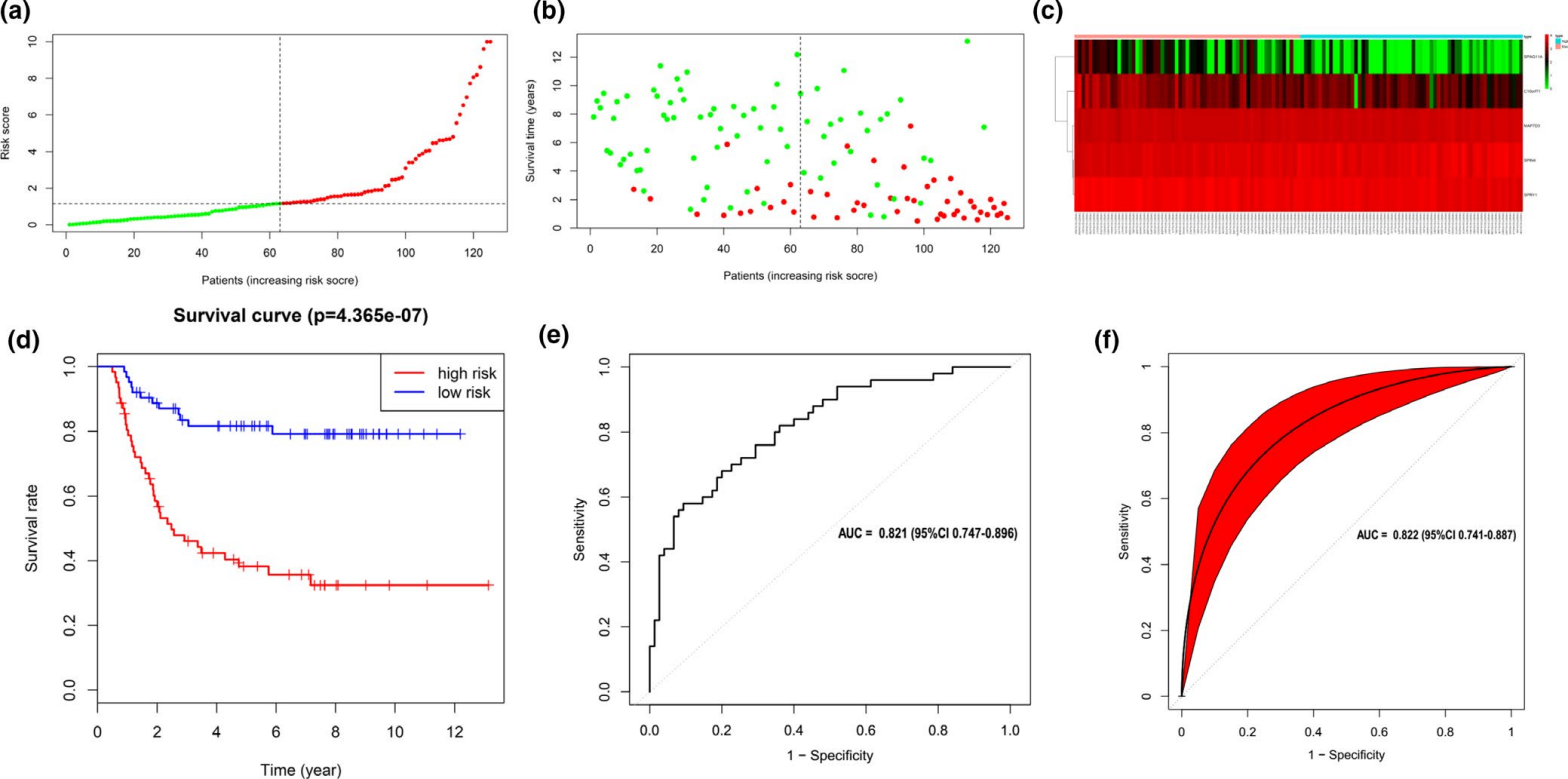
Prognostic evaluation of the five‐mRNA signature in pediatric Wilms tumor patients. (a) The distribution of mRNA‐related survival risk score. (b) The distribution of mRNA‐related survival time. (c) Gene expression heatmap of five identified genes between high‐risk and low‐risk groups. (d) Kaplan–Meier survival curve analysis for overall survival of pediatric Wilms tumor patients between low‐ and high‐risk groups. (e) Receiver operating characteristic (ROC) curve indicated that the area under receiver operating characteristic of 5‐mRNA model was 0.821. (f) Bootstrap test with 500 times was used to perform the internal validation indicated that the area under receiver operating characteristic of 5‐mRNA model was 0.822

For each patient, a risk score analysis was conducted among the five mRNAs to determine the risk score (Table 1). The risk score formula is as follows: Risk score = 0.3036*SPIN4 + 0.8576*MAP7D3 −0.1548*C10orf71 −0.7335*SPRY1 −0.2654*SPAG11A. The distribution of survival risk score of these five mRNAs and mRNA‐related survival time were demonstrated in Figures 2a,b. The expression Heatmap of five‐mRNA signature was demonstrated in Figure 2c. The pediatric WT patients were divided into low‐risk group and high‐risk group based on the median risk score (value = 1.1503). Survival analysis between high‐risk group and low‐risk group was performed by using the log‐rank test (Figure 2d). The result revealed that low‐risk group was related to a better prognosis (*p* < .001).

The results of ROC demonstrated that AUC was 0.821 (95%CI [0.747, 0.896]) (Figure 2e). We have used bootstrap test (Bootstrap resampling times = 500) to perform the internal validation. The results demonstrated that the validated AUC was 0.822 (95%CI [0.741, 0.887]) (Figure 2f), which was consistent with primary results of AUC (0.821). The results demonstrated that the 5‐mRNA prognostic model had a promising sensitivity and specificity in predicting the survival outcomes of pediatric WT patients.

### 3.4 REACTOME, KEGG, and BIOCARTA pathway analyses among survival‐related mRNAs

We then included 466 survival‐related mRNAs screened by univariate Cox analysis (*p* < .05) into pathway analyses. A total of 47 pathway ways were enriched in this study, including 16 pathways enriched by KEGG database, 29 pathways enriched by REACTOME database, and 2 pathways enriched by BIOCARTA database. The top five enriched pathways ranked as the P value were demonstrated in Table 2. The results demonstrated that these survival‐related genes were mainly enriched in ErbB2 and ErbB3 signaling pathways, and calcium signaling pathway.

**Table 2:** The results of pathway analyses including REACTOME, KEGG, and BIOCARTA pathway databases

Category	Term	Count	*p* Value	Genes	FDR
REACTOME	R‐HSA‐1250196: SHC1 events in ERBB2 signaling	5	9.62E‐04	*NRAS, ERBB3, ERBB2, EGF, NRG1*	1.350958906
REACTOME	R‐HSA‐419408: Lysosphingolipid and LPA receptors	4	0.004144594	*S1PR1, PLPPR4, PLPPR5, LPAR1*	5.702267239
REACTOME	R‐HSA‐1306955: GRB7 events in ERBB2 signaling	3	0.005525286	*ERBB3, ERBB2, NRG1*	7.533745676
REACTOME	R‐HSA‐1963640: GRB2 events in ERBB2 signaling	4	0.006153572	*NRAS, ERBB2, EGF, NRG1*	8.356172174
REACTOME	R‐HSA‐1963642: PI3K events in ERBB2 signaling	4	0.006153572	*ERBB3, ERBB2, EGF, NRG1*	8.356172174
KEGG	hsa04020: Calcium signaling pathway	12	0.002926977	*EDNRA, ADCY7, CHRM2, ERBB3, ERBB2,* *PHKA1, CACNA1G, PPP3CA, NTSR1, GRM1,* *CACNA1A, F2R*	3.649269099
KEGG	hsa05202: Transcriptional misregulation in cancer	11	0.005402032	*PLAT, PRCC, RXRG, MDM2, IGF1, BCL6,* *WHSC1, NGFR, ZBTB16, HIST2H3D, MYCN*	6.638940309
KEGG	hsa05200: Pathways in cancer	18	0.009581263	*BMP4, ADCY7, ERBB2, RXRG, TGFB3, IGF1,* *LPAR1, ZBTB16, MECOM, FZD7, EDNRA, CCNE1,* *NRAS, CCDC6, CBLB, MDM2, EGF, F2R*	11.49385516
KEGG	hsa04068: FoxO signaling pathway	9	0.012608779	*NRAS, S1PR1, PRKAB2, TGFB3, MDM2,* *IGF1, BCL6, EGF, GRM1*	14.86439542
KEGG	hsa05215: Prostate cancer	7	0.016293117	*CCNE1, NRAS, ERBB2, MDM2, IGF1, PDGFC, EGF*	18.80657177
BIOCARTA	ErbB3 pathway	3	0.008074119	*ERBB3, EGF, NRG1*	8.793866109
BIOCARTA	EGFR/SMRTE pathway	3	0.039655032	*THRA, ZBTB16, EGF*	36.83536607

## 4 DISCUSSION

Evidence has proved that mRNAs play crucial roles in the tumorigenesis and progression of pediatric WT (Apelt et al., 2016; Martins, Pinto, Domingues, & Cavaco, 2018; Zhu et al., 2018). Although several previous studies have identified several mRNAs with prognostic value in pediatric WT, they were not focused on the correlations between mRNA signature model and prognosis of pediatric WT (Gadd et al., 2017; Ludwig et al., 2016; Wari et al., 2017). Moreover, with the development of detection technology, the single mRNA expression pattern was no longer sufficient for accurate predication of prognosis of pediatric WT.

To the best of our knowledge, it is the first time to screen out the DEGs between pediatric WT and paired tissues from TARGET database. A novel five‐mRNA signature (*SPRY1*, *SPIN4*, *MAP7D3, C10orf71*, and *SPAG11A*) was then generated. ROC curve proved that this five‐mRNA signature revealed a high sensitivity and specificity in predicating the survival outcomes of pediatric WT patients. The predictive value of the five‐mRNA signature was validated in TARGET dataset of 136 pediatric WT patients. Based on these five prognostic mRNAs, we established a five‐mRNA prognostic model which can classify pediatric WT patients into low‐risk and high‐risk groups with different survival outcomes.

Wilms tumor are most common types of childhood kidney cancers. It has been reported that for children younger than 15 years with Wilms tumor, the 5‐year survival rate has increased over the same time from 74% to 88% (Smith, Altekruse, Adamson, Reaman, & Seibel, 2014). The 5‐year survival rate for Wilms tumor with favorable histology has been consistently above 90% since the 1980s (Smith, Altekruse, Adamson, Reaman, & Seibel, 2014). The results of this manuscript demonstrated that among these five mRNAs, *SPRY1* and *SPIN4* were associated with high risk of development of pediatric WT, and *MAP7D3, C10orf71*, and *SPAG11A* were associated with low risk of development of pediatric WT.

In mammals, *SPRY1* was reported to be consisted of four members and was inhibitor of receptor tyrosine kinase signaling (Rozen et al., 2009). In mice, *SPRY1* plays an important role during kidney morphogenesis by antagonizing GDNF signaling (Basson et al., 2005). *SPRY1* also plays an important role in the early steps of glomerulus formation and represents a physiologically associated target gene of *WT1* during the development of kidney (Gross et al., 2003). *SPRY1* was reported to be associated with many kinds of tumors, such as breast cancer (He et al., 2016), colorectal cancer (Zhang et al., 2016), and human epithelial ovarian cancer (Masoumi‐Moghaddam, Amini, Wei, Robertson, & Morris, 2015). The protein encoded by *MAP7D3* belongs to the *MAP7* family. There is little known about the role of *MAP7* with respect to cancer progression (Blum et al., 2008). Many important cellular processes attributed to microtubules involvement, including cell division, motility, and changes in cell shape (Bhat & Setaluri, 2007). Yan et al. (Yan et al., 2013) showed that miR‐16 targeting *MAP7* played an important role in regulating proliferation in cancer cells. Also, Blum et al. (Blum et al., 2008) demonstrated that the expression ratio of *MAP7/B2M* can be regarded as a prognostic factor for survival in patients with colon cancer. Peng et al. (Lin et al., 2019) demonstrated that *SPAG11A* was involved in the biological process of papillary thyroid cancer. However, the *SPIN4 and C10orf71* have not been reported associated with the development and progression of cancer. To the best of our knowledge, given the potential molecular mechanism of the five mRNAs signature, no reports of the function and mechanism of these five mRNAs, *SPRY1*, *SPIN4*, *MAP7D3, C10orf71*, and *SPAG11A*, have been published concerning WT.

The development of pediatric WT is a multi‐step process. A large number of genetic alterations were involved in this multi‐step biological process (Morrison, Viney, Saleem, & Ladomery, 2008). For the sake of elucidating the effects and functions of these survival‐related mRNAs screened by univariate Cox analyses, we used REACTOME, KEGG, and BIOCARTA pathway databases to perform pathway analyses. The results demonstrated that these survival‐related genes were mainly enriched in *ErbB2* and *ErbB3* signaling pathways and calcium signaling pathway.

Both BIOCARTA and REACTOME pathway databases revealed that these survival‐related genes were mainly enriched in *ErbB2* and *ErbB3* signaling pathways. *ErbB2* and *ErbB3* belong to the family of human epidermal growth factor receptors consisting of *EGFR* (*ErbB1*), *ErbB2*, *ErbB3*, and *ErbB4* (Vermeulen, Segers, & De Keulenaer, 2016). *ErbB2* amplification plays a critical role in tumor growth. Amplified *ErbB2* can bind to *ErbB3* to form an oncogenic *ErbB2/ErbB3* complex (Holbro et al., 2003). *ErbB3* interacts with the regulatory p85 subunit of PIK3 in this complex to activate the PI3K/Akt pathway and intense cell growth and proliferation. (Schoeberl et al., 2009) Therefore, *ErbB3* plays an important role in oncogenic *ErbB2* signaling pathway. Rotter et al. (Rotter, Block, Busch, Thanner, & Hofler, 1992) reported that the expression of *ErbB2* was downregulated in the renal cell carcinoma when compared with normal kidney tissue. To the best of our knowledge, the molecular mechanisms behind the alteration of *ErbB2* in renal cell carcinoma compared with normal kidney was still unknown. Plus, the expression of *ErbB3* has not been thoroughly studied in renal cell carcinoma. KEGG pathway database revealed that these survival‐related mRNAs were mainly enriched in calcium signaling pathway. Previous studies (Cole & Kohn, 1994; Soboloff, Zhang, Minden, & Berger, 2002; Sukumaran, Sun, Vyas, & Singh, 2015) have been reported that inhibition of calcium influx can cause either growth arrest or cell death in numbers of cancer cells. However, the role of calcium signaling pathway in the development and progression of WT has not been elucidated yet. Xu et al. (Xu, Chen, Ye, Zhong, & Chen, 2015) reported that calcium signaling pathway has been involved in inducing the apoptosis in non‐small cell lung cancer cells, for the overload of calcium has been reported to play a crucial role in the initiation and regulation of apoptosis.

There are some limitations in this study. The predictive value of the five‐mRNA signature was not validated in another independent dataset because it is very difficult for us to obtain tumor specimens, especially pediatric tumor samples.

## 5 CONCLUSION

In conclusion, the five‐mRNA signature can predict the prognosis of patients with pediatric WT. It has significant implication in the understanding of therapeutic targets for pediatric WT patients. However, further study is needed to validate this five‐mRNA signature and uncover more novel diagnostic or prognostic mRNA candidates in pediatric WT patients.

## CONFLICT OF INTEREST

The authors declare no conflicts of interest.

## AUTHOR CONTRIBUTION

Study conception and design: Xiao‐Dan Lin; Yong Wei; Qing‐Shui Zheng; Xue‐Yi Xue; Ning Xu. Data acquisition: Zhi‐Bin Ke; Dong‐Ning Chen; Yun‐Zhi Lin. Analysis and data interpretation: Xiao‐Dan Lin; Yu‐Peng Wu; Zhi‐Bin Ke; Xiong‐Lin Sun; Xiao‐Dong Li. Drafting of the manuscript: Xiao‐Dan Lin; Ning Xu; Yu‐Peng Wu; Shao‐Hao Chen; Xiong‐Lin Sun. Critical revision: Ning Xu; Yu‐Peng Wu; Shao‐Hao Chen; Qing‐Shui Zheng; Xue‐Yi Xue.

## ETHICAL STATEMENT

Not applicable.
